# Cosolvent-Driven Interfacial Polymerization for Superior Separation Performance of Polyurea-Based Pervaporation Membrane

**DOI:** 10.3390/polym13081179

**Published:** 2021-04-07

**Authors:** Manuel Reyes De Guzman, Micah Belle Marie Yap Ang, Shu-Hsien Huang, Fang-Chi Hu, Yu-Hsuan Chiao, Hui-An Tsai, Kueir-Rarn Lee

**Affiliations:** 1 Material Corrosion and Protection Key Laboratory of Sichuan Province, School of Materials Science and Engineering, Sichuan University of Science and Engineering, Zigong 643000, China; manuelrdg@yahoo.com; 2 R&D Center for Membrane Technology and Department of Chemical Engineering, Chung Yuan Christian University, Taoyuan 32023, Taiwan; mbmyang@gmail.com (M.B.M.Y.A.); huian@cycu.edu.tw (H.-A.T.); krlee@cycu.edu.tw (K.-R.L.); 3 Department of Chemical and Materials Engineering, National Ilan University, Yilan 26047, Taiwan; fangci@hotmail.com; 4 Department of Chemical Engineering, University of Arkansas, Fayetteville, AR 72701, USA; ychiao@uark.edu; 5 Research Center for Circular Economy, Chung Yuan Christian University, Taoyuan 32023, Taiwan

**Keywords:** thin-film composite membranes, pervaporation, interfacial polymerization, polyurea, membrane separation

## Abstract

A thin-film composite (TFC) polyurea membrane was fabricated for the dehydration of an aqueous tetrahydrofuran (THF) solution through interfacial polymerization, wherein polyethyleneimine (a water-soluble amine monomer) and m-xylene diisocyanate (an oil-soluble diisocyanate monomer) were reacted on the surface of a modified polyacrylonitrile (mPAN) substrate. Cosolvents were used to tailor the membrane properties and increase the membrane permeation flux. Four types of alcohols that differed in the number of carbon (methanol, ethanol, isopropanol, and tert-butanol) were added as cosolvents, serving as swelling agents, to the aqueous-phase monomer solution, and their effect on the membrane properties and pervaporation separation was discussed. Attenuated total reflection Fourier transform infrared spectroscopy confirmed the formation of a polyurea layer on mPAN. Field emission scanning electron microscopy and surface water contact angle analysis indicated no change in the membrane morphology and hydrophilicity, respectively, despite the addition of cosolvents for interfacial polymerization. The TFC membrane produced when ethanol was the cosolvent exhibited the highest separation performance (permeation flux = 1006 ± 103 g·m^−2^·h^−1^; water concentration in permeate = 98.8 ± 0.3 wt.%) for an aqueous feed solution containing 90 wt.% THF at 25 °C. During the membrane formation, ethanol caused the polyurea layer to loosen and to acquire a certain degree of cross-linking. The optimal fabrication conditions were as follows: 10 wt.% ethanol as cosolvent; membrane curing temperature = 50 °C; membrane curing time = 30 min.

## 1. Introduction

Interfacial polymerization refers to the formation of a thin dense layer on a substrate or a supporting layer from the reaction between highly reactive immiscible monomers, which both dissolve in a pair of immiscible solvents. These two immiscible phases commonly contain water in one phase and an organic solvent in the other. In the water phase, the monomer is usually an amine type, while in the water-immiscible organic phase, the usual monomer is acyl chloride or diisocyanate ester. Some main advantages of interfacial polymerization are as follows: rapid reaction at room temperature, no need to consider the stoichiometric amounts of reactants, and no requirement for high purity of reactants. Interfacial polymerization is suitable for preparing thin-film composite membranes (TFC), consisting of an ultrathin and dense separation layer deposited on a porous substrate. A high flux is delivered by the very thin layer (the porous substrate does not affect the flux but only serves as membrane support, giving mechanical strength to the layer), and the layer denseness ensures high selectivity [1,2].

TFC membranes manufactured through the method of interfacial polymerization have been widely used in the fields of reverse osmosis [3,4] and nanofiltration [5,6,7] separation processes, but are rarely applied to pervaporation. Interfacial polymerization produces a variety of polymer layers, including those of polyamide [8,9,10,11,12], polyester [8,9,13,14,15,16,17], and polyurea [18]. In 1977, Rozelle et al. [19] used polyethyleneimine (PEI) and toluene diisocyanate (TDI) for interfacial polymerization to prepare NS-100 composite polyurea membrane. In the same year, polyurea and TDI were considered for polymerization reaction to produce RC100 composite polyurea membrane. Both aforementioned composite membranes were applied in reverse osmosis separation processes. In 1999, Zhao et al. [20] prepared a polyurethane-urea (PUU) membrane with a dual-soft segment and applied it to the pervaporation separation of an aqueous ethanol solution. In 2006, DAS et al. [21] made a series of interpenetrating network (IPN) films composed of hydroxy-terminated polybutadiene-based PUU and poly(methyl methacrylate) membranes, and used them to separate the components of an aqueous dimethylformamide (DMF) solution. They found that the series of IPN films had a preference for the permeation of DMF. The main reason was the small dissolution parameter difference between the films and DMF (in other words, the films had a higher affinity with DMF). In 2006, Liu et al. [22] employed m-phenylenediamine (MPD) and 5-isocyanato-isophthaloyl chloride (ICIC) to prepare composite polyamide-urea reverse osmosis membranes and explored the anti-fouling ability. They discovered that, compared with composite trimesoyl chloride-MPD and energy-saving polyamide membranes, ICIC-MPD had a better anti-fouling ability. In 2008, Das et al. [23] used porous PUU membranes to separate aqueous phenol and chlorophenol solutions. The results of their study demonstrated that, compared with dense PUU membranes, porous PUU membranes had better separation performance. In 2010, Sadeghi et al. [24] discussed the influence of urethane and urea content in PUU membranes on gas separation efficiency.

In this study, a TFC polyurea membrane was manufactured by means of interfacial polymerization, wherein an amine monomer (PEI) (water-phase monomer) and a diisocyanate monomer (m-xylene diisocyanate, XDI) (organic-phase monomer) were reacted on a modified polyacrylonitrile (mPAN) support to produce a TFC polyurea membrane for the dehydration of an aqueous tetrahydrofuran (THF) solution. In pharmaceutical laboratories and industries, THF is a common solvent for polar and nonpolar compounds and is often used in organic synthesis. However, THF with high purity commands a high unit price, so dehydration and recycling of THF not only save cost but also protect the ecological environment. THF and water form an azeotropic mixture (composition is 5.3 wt.% water and 94.7 wt.% THF). The dehydration of aqueous THF solutions by using traditional distillation is not easy to achieve; moreover, it consumes energy and has a high economic cost. On the other hand, the use of the pervaporation separation process for the purpose of achieving effective dehydration of aqueous THF solutions saves energy, is not restricted by gas-liquid equilibrium, and can effectively break the azeotropic point of THF and water [25,26,27].

Our present work intends to explore the potential of TFC polyurea membranes fabricated through interfacial polymerization in the uncharted field of pervaporation (interfacial polymerization is generally used for reverse osmosis and nanofiltration processes); moreover, we integrated for the first time the technique of introducing a cosolvent into interfacial polymerization for such an application to improve the membrane performance. A cosolvent acts as a swelling agent that makes polymer chains soft, so they move with more freedom; as a result, the polymer is easy to process and modify [28,29,30,31]. Alcohols are an example of swelling agents, and their added advantages are that they dissolve in amine monomers and are miscible with toluene. Hence, we chose alcohols as a cosolvent in the aqueous-phase solution for interfacial polymerization (or as an agent to swell the membrane substrate), as they mix well with water. Adding alcohols as a cosolvent to the water-phase solution had two functions: (1) when the asymmetric substrate was immersed in the aqueous amine solution, the alcohol could swell the substrate, thereby more amine monomers could diffuse and be retained in the substrate; (2) when the substrate previously immersed in the water-phase solution was contacted with the organic-phase solution to initiate interfacial polymerization, the alcohol served as a carrier, bringing with it amine monomers, as it blended with the organic-phase solution. Therefore, more diisocyanate monomers were able to react with the amine, forming a polyurea layer with a looser structure (larger free volume). This effect would be beneficial to the improvement of the membrane permeation flux for a more efficient pervaporation separation process.

## 2. Methods

### 2.1. Experimental Chemicals

The following chemicals were used, along with the description and the source company: polyacrylonitrile (PAN), Donghua Synthetic Fiber Co. Ltd., Taipei, Taiwan; 1-methyl-2-pyrrolidone (NMP), reagent grade, Tedia Company Inc. (Fairfield, OH, USA); sodium hydroxide (NaOH), reagent grade, Fullin Chemical Co. Ltd., Taipei, Taiwan; PEI, water-phase amine monomer, reagent grade, Alfa Aesar, Haverhill, MA, USA; XDI, organic-phase diisocyanate monomer, Tokyo Chemical Industry Co. Ltd. Tokyo, Japan; distilled water, aqueous-phase solvent, produced in the laboratory; toluene, organic solvent, reagent grade, Echo Chemical Co. Ltd., Miaoli, Taiwan; methanol, reagent grade, Mallinckrodt, Staines-upon-Thames, United Kingdom; ethanol, reagent grade, Jingming Chemical Co. Ltd.; 2-propanol or isopropanol (IPA), reagent grade, Echo Chemical Co. Ltd., Miaoli, Taiwan; tert-butyl alcohol, reagent grade, Scharlau, Barcelona, España; THF, reagent grade, Echo Chemical Co. Ltd., Miaoli, Taiwan; and liquid nitrogen, Zhenghong Gas Co. Ltd., Yilan, Taiwan.

### 2.2. Preparation of Substrate

PAN was dissolved in NMP, and a 15 wt.% casting polymer solution was prepared. At room temperature, the solution was fully stirred for homogenization by using a magnetic stirrer. Then, the casting solution was placed at room temperature for one day to remove the bubbles caused by stirring. A wet-phase inversion method was adopted, wherein the casting solution was spread and cast on nonwovens with the use of a casting knife, and then the cast film was immersed in a coagulation tank (the coagulant in this process was water). After the polymer solidified, the coagulant in the tank was changed many times to ensure full and efficient exchange between the solvent and the coagulant. Finally, the PAN substrate was stored in distilled water.

To improve the hydrophilicity of the substrate so that aqueous amine solution could be more easily distributed on the surface, PAN was chemically modified. The PAN substrate was soaked in 2 M aqueous NaOH solution at 50 °C for hydrolysis that converted part of the –CN functional groups on the PAN polymer chains into hydrophilic –COOH or –CONH_2_ [32]. After the hydrolysis modification, the hydrolyzed PAN (mPAN) substrate was soaked in distilled water, washed for several hours, and stored in distilled water.

### 2.3. Preparation of Composite Membrane

**Figure 1:**
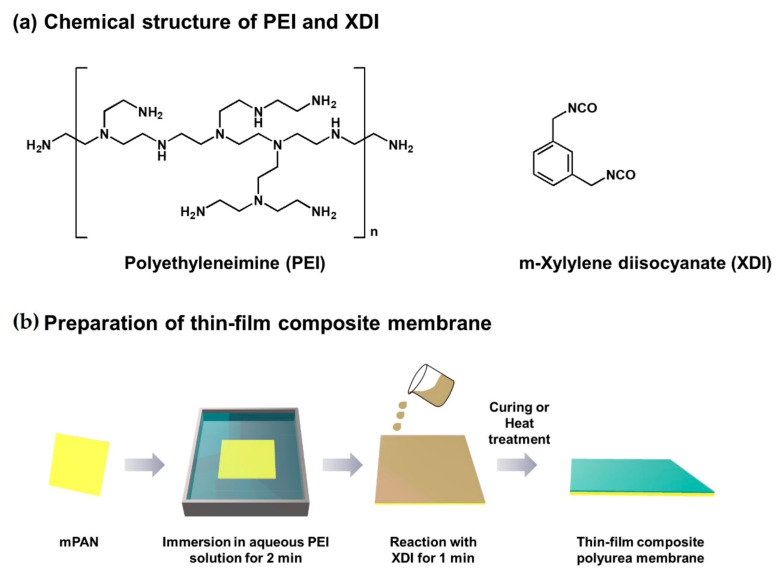
(**a**) Chemical structure of monomers; (**b**) preparation of thin-film composite (TFC) membrane.

XDI, an organic-phase monomer, was dissolved in toluene and stirred evenly at room temperature to prepare a 1 wt.% organic-phase solution. The chemical structure of XDI is shown in Figure 1. In this study, alcohols with different carbon numbers (methanol, ethanol, isopropanol, and tert-butanol) were used as cosolvents to serve as swelling agents, and they were dissolved in distilled water and stirred at room temperature to prepare a 10 wt.% aqueous alcohol solution. PEI, an aqueous monomer, was dissolved in distilled water or in 10 wt.% aqueous alcohol solution, and the solution was stirred well at room temperature to prepare a 1 wt.% aqueous solution. The chemical structure of PEI is also shown in Figure 1. The substrate (mPAN) was soaked in the aqueous solution for 2 min. The excess aqueous solution on the surface of the substrate was removed gently with a glass rod, and then the surface was contacted with the organic solution for 1 min. With the system having immiscible aqueous and organic phases, an interfacial polymerization reaction between PEI and XDI was carried out on the surface of mPAN to form a polyurea layer. The composite film was dried at room temperature for 1 min. Afterward, the film was heat-treated or cured in an oven at 50 °C for 30 min to improve the stability of the polyurea layer (i.e., to make it more firm or integrated and to ensure it completely adhered to the substrate). The composite polyurea membrane was soaked in methanol for cleaning and to remove the unreacted monomer, and it was finally dried in a vacuum oven prior to use.

### 2.4. Evaluation of Membrane

#### 2.4.1. Field Emission Scanning Electron Microscopy

To observe the membrane morphology, field emission scanning electron microscopy (FESEM, S-4800, Hitachi Co., Tokyo, Japan) images were taken. The image resolution was extremely high, and the magnification reached tens of thousand times. A sample was cut into a small piece, frozen in liquid nitrogen, torn, and fixed on a stage with carbon tape. Then, a layer of Pt/Pb metal layer was evaporated in a device under vacuum to make the sample placed in the device become a conductor, and this acquired property would reduce the surface discharge effect of the sample. The metal layer could also prevent the polymer sample from cracking during FESEM analysis due to high-speed electron beam irradiation.

#### 2.4.2. Attenuated Total Reflection Fourier Transform Infrared (ATR-FTIR) Spectrometry

The chemical structure of membrane samples was analyzed through attenuated total reflection Fourier transform infrared (ATR-FTIR, Perkin Elmer Spectrum 100 FTIR Spectrometer, Waltham, MA, USA) spectrometry. IR spectroscopy was used in the mid-infrared region, which ranged from 4000 to 400 cm^−1^, for qualitative and quantitative analyses. Its main function was to identify organic compounds. The principle is that after IR light is used to irradiate the sample, the spectrum generated would have different characteristics due to the different absorption energies required for molecular vibration or rotation. The chemical structure and bonding type in the sample to be measured can be obtained based on the characteristic spectrum.

#### 2.4.3. Contact Angle Measurement

The membrane was cut into the required size and placed on the contact angle measuring instrument (Kyowa Interface Science Co. Ltd., Niiza-City, Saitama, Japan). Deionized water as the test liquid was dropped onto the membrane surface. The change in water contact angle on the membrane surface was observed, and then hydrophilic properties were analyzed. The larger the water contact angle, the higher the hydrophobicity of the membrane; on the contrary, the smaller the water contact angle, the higher the hydrophilicity of the membrane.

#### 2.4.4. Pervaporation Test

The composite polyurea membrane prepared in this study was applied to the pervaporation separation of a 90 wt.% aqueous THF solution at 25 °C. The pervaporation test was similar to that in our previous work [33]. Two stainless steel disks constituted the pervaporation chamber; the top disk formed the upper chamber, and the bottom disk the lower chamber. The feed solution was in direct contact with the polyurea layer, and the composite membrane was supported by a filter paper and a highly porous sintered copper plate. An O-ring in between the pervaporation upper and lower chambers held the membrane in place, making sure it was in a tight state. An effective membrane area of 7.07 cm^2^ was fixed. The downstream pressure was 1.0 cmHg, and the operating temperature (feed solution temperature) was 25 °C. After the machine was turned on and stabilized, the sample was taken (sampling). A trap with liquid nitrogen was used to freeze and collect the permeated components. After the permeate was completely melted, the permeation flux was calculated using a gravimetric method, wherein the weight of the trap before and after sampling was recorded. The concentrations of feed and permeate were determined using gas chromatography (China Chromatography GC-2000, Taipei, Taiwan). A 0.2 µL feed or permeate was injected into the gas chromatography column to analyze its composition. The permeation flux and the separation factor for the membrane were calculated by the following formula:

(1) $$Q=\frac{W}{A\times t}$$

where *Q* was the permeation flux (g/m^2^h); *W* was the weight of water that passed through the membrane (g); *A* was the effective membrane area (m^2^); and *t* was the operation time (h).

(2) $$\alpha_{A/B}=\frac{\frac{Y_{A}}{Y_{B}}}{\frac{X_{A}}{X_{B}}}$$

where *Y_A_* and *Y_B_* were the concentrations of water and THF in the permeate and *X_A_* and *X_B_* were the concentrations of water and THF in the feed.

## 3. Results and Discussion

### 3.1. Characterization of Membrane

**Figure 2:**
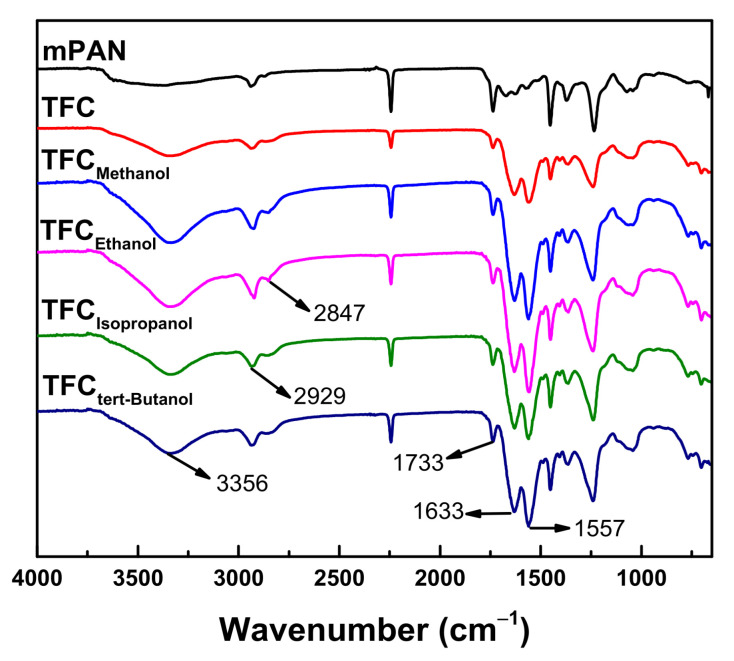
ATR-FTIR spectra of modified polyacrylonitrile (mPAN) and TFC membranes.

Figure 2 depicts ATR-FTIR spectra of mPAN support and TFC membranes. For TFC membranes, wavenumbers at 1733 and 1633 cm^−1^ represented carbonyl group, C=O. The peak at 3356 cm^−1^ was related to the NH (secondary amine) stretching in PEI, while the peak at 1557 cm^−1^ described N-H bond, which was connected to the carbonyl group in polyurea. The bond at 2929 and 2847 cm^−1^ in polyurea represented C-H bonding in methylene and methyl stretching, respectively [34,35]. Therefore, a polyurea thin-film layer was formed on top of the mPAN support.

**Figure 3:**
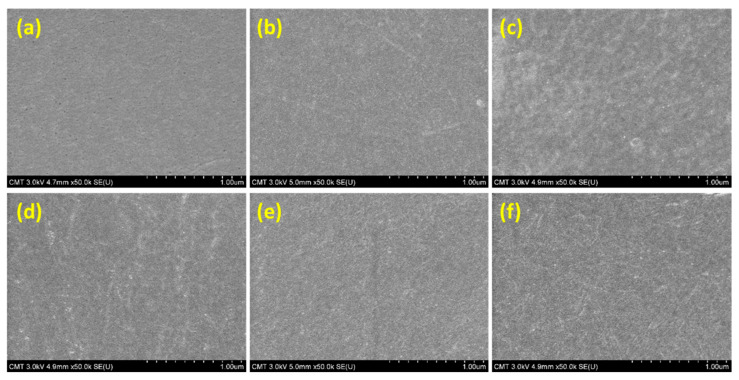
Surface FESEM images: (**a**) mPAN; (**b**) TFC; (**c**) TFC_Methanol_; (**d**) TFC_Ethanol_; (**e**) TFC_Isopropanol_; and (**f**) TFC_tert-Butanol_.

A number of studies reported that when a cosolvent was used, the surface morphology of the thin-film layer formed through interfacial polymerization would vary. However, in our system, the polyurea surface (Figure 3) did not change when a cosolvent was used. All membranes obtained after conducting interfacial polymerization on an mPAN support had a smooth surface without nodules. This denseness of the polyurea surface was responsible for the high separation efficiency of the membrane.

Changing the type of cosolvent possibly affects the water contact angle of membranes (Table 1). TFC had a contact angle of 63.55 ± 2.07°; however, when a methanol or ethanol cosolvent was used, the contact angle of TFC_Methanol_ and TFC_Ethanol_ turned out to be 61.40 ± 3.87 and 63.96 ± 5.18°, respectively. The modified membranes obtained from using a methanol or ethanol cosolvent had somewhat lower or similar contact angles, probably because the hydrophilic segments of polyurea were facing toward the membrane surface. However, when the cosolvent used was isopropanol or tert-butanol, the contact angle of the resultant modified membranes was relatively higher (66.31 ± 2.57 for TFC_Isopropanol_ and 67.40 ± 2.86° for TFC_tert-Butanol_).

The thickness of the polyurea layer deposited on mPAN may vary, depending on the type of cosolvent introduced into the aqueous phase that participated in interfacial polymerization. To examine the top layer thickness in TFC membranes, cross-sectional images at a high magnification are given in Figure S1. However, the images revealed that the selective layer was not thick enough (this result may serve as an indication that the fabricated TFC membrane consisted of an ultrathin top layer). As such, we were unable to observe any probable changes that might have occurred. Hence, conclusions could not be drawn about the possible effect of the cosolvent on the thickness of the polyurea selective layer.

### 3.2. Identification of Best Cosolvent

**Figure 4:**
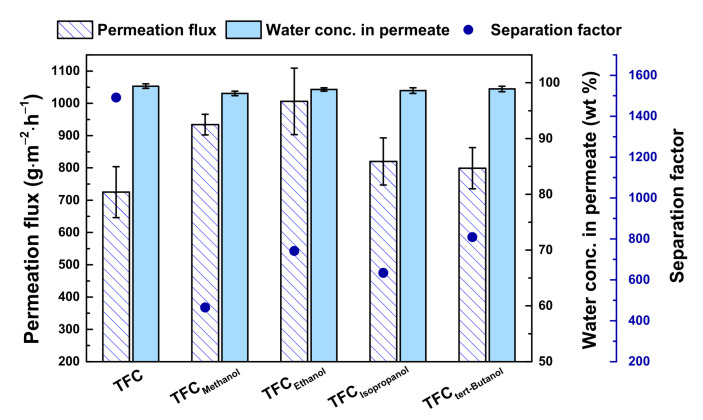
Separation performance of TFC membranes. Feed = 90 wt.% aqueous tetrahydrofuran (THF) solution; feed temperature = 25 °C. Interfacial polymerization conditions: 10 wt.% cosolvent; membrane curing temperature = 50 °C; membrane curing time = 30 min.

Figure 4 compares the membrane performance. The data was for a feed of 90 wt.% THF solution at 25 °C. All modified membranes had higher permeation flux than the pristine membrane. The water concentration in the permeate was maintained. This was because the cosolvent at a certain concentration helped loosen the polyurea thin-film. The cosolvent transferred quicker to the interface and met toluene. Upon their encounter, a layer of polyurea formed faster.

Some alcohol became trapped in the polyurea layer, and during the membrane curing, it would evaporate and leave behind some free volume. The molar volume of alcohol molecules increased in the following order: methanol (40.7 mL·mol^−1^) < ethanol (58.6 mL·mol^−1^) < isopropanol (76.8 mL·mol^−1^) < tertiary butanol (91.9 mL·mol^−1^) [36,37]. Compared with the permeation flux for TFC_Methanol_ (934 ± 32 g·m^−2^·h^−1^), that for TFC_Ethanol_ (1006 ± 103 g·m^−2^·h^−1^) was higher because ethanol had larger chains and molar volume than methanol. It was possible that during evaporation, ethanol left behind a larger free volume. However, for the case of isopropanol or tertiary butanol, whose molar volume was larger than either methanol or ethanol, the permeation flux for TFC_Isopropanol_ (820 ± 73 g·m^−2^·h^−1^) and TFC_tert-Butanol_ (799 ± 64 g·m^−2^·h^−1^) turned out to be lower than that for TFC_Ethanol_. At the same concentration (10 wt.%) of alcohols in the solution, the molar concentration of isopropanol or tert-butanol was less than that of methanol or ethanol, which is in accordance with the comparison of molar volumes of the alcohols. In other words, the amount of isopropanol or tert-butanol was less because either of them had a relatively higher molar volume than that of methanol or ethanol. It would follow then that a lower amount of isopropanol or tert-butanol became stuck in the polyurea layer, which means that smaller and fewer number of free volume would be created in the polyurea layer; thereby, a low permeation flux was obtained.

### 3.3. Optimum Conditions for Composite Membrane Manufactured via the Best Cosolvent

**Figure 5:**
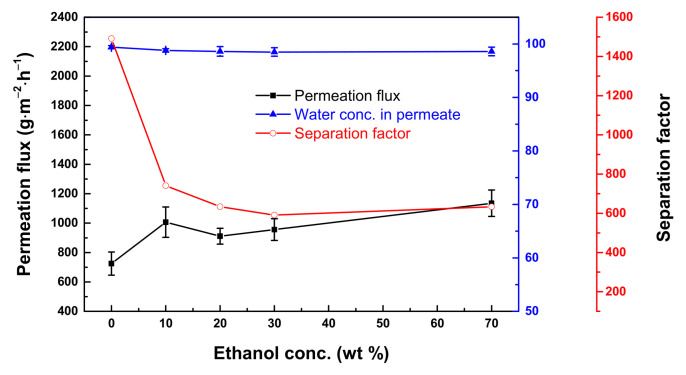
Effect of concentration of ethanol added to the aqueous phase on the membrane performance. Feed = 90 wt.% aqueous THF solution; feed temperature = 25 °C. Interfacial polymerization conditions: membrane curing temperature = 50 °C; membrane curing time = 30 min.

Because the best cosolvent was ethanol, we varied the ethanol concentration from 10 to 70 wt.%. At a concentration greater than 10 wt.% ethanol, the membrane permeation flux remained the same (Figure 5). An increase in the cosolvent concentration would bring about swelling of the membrane support. Therefore, the amount of amine adsorbed was greater. If there was more amine on the membrane surface, the reaction would be quicker; thus, the formation of the first layer of a dense polyamide was also quicker. This phenomenon is similar to the self-limiting effect. That is, if the first layer is formed, other amines would not be able to penetrate through the interface to react with XDI. Therefore, the polyurea layer had reached its densest form. Therefore, the permeation flux remained the same.

**Figure 6:**
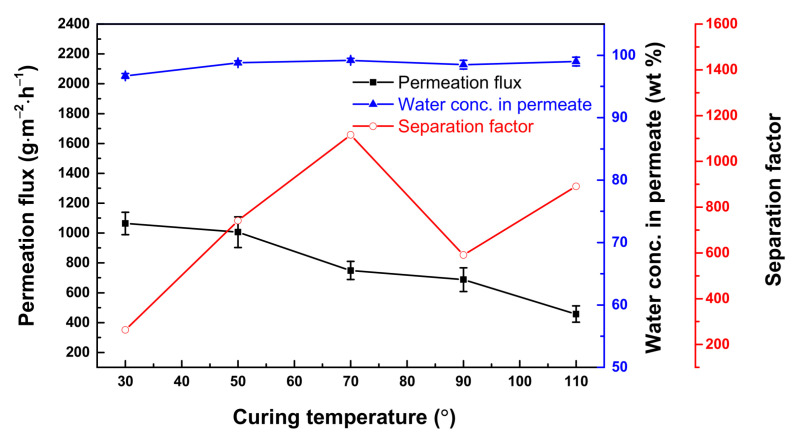
Effect of curing temperature on membrane performance. Feed = 90 wt.% aqueous THF solution; feed temperature = 25 °C. Interfacial polymerization conditions: 10 wt.% ethanol; membrane curing time = 30 min.

Figure 6 presents the membrane performance vs. the curing temperature (30–110 °C). The purpose of curing the membrane was for further polymerization of the monomers (thereby densifying the membrane to a higher level) and to remove any residual toluene from the membrane [38]. In this case, the isocyanate group in the polyurea membrane acted as a facilitator, allowing water molecules to pass through it. Unreacted isocyanate groups of XDI would react with water molecules to form unstable intermediate amino carbonic acid. This amino carbonic acid would react further with alkyl amine radicals of PEI to form urea linkage [18,39,40]. Moreover, curing or heat treatment may also cause charge-transfer-complex in polyurea, which is an intra- and inter-molecular interaction between the benzene rings (electron-donating groups), resulting in a more compact layer [41].

From 30 to 110 °C, the permeation flux decreased from 1064 ± 75 to 457 ± 55 g·m^−2^·h^−1^. The water concentration in the permeate reached 98.8 ± 0.3 wt.% (α_A/B_ = 741) when the curing temperature was 50 °C. At a temperature higher than 50 °C, the change in water concentration in the permeate was not significant. At 30 °C, the water concentration in the permeate was only 96.7 ± 0.4 wt.% (α_A/B_ = 269) because the temperature was not high enough to densify the membrane. Figure S2 shows changes in the membrane morphology when the curing temperature was raised from 30 to 110 °C. At a temperature higher than 70 °C, the membrane surface displayed a nodular structure. The reaction rate was probably enhanced at high temperatures, and this caused the formation of nodules on the surface. Such a nodular structure might be associated with microscopic porosity on the surface. Nevertheless, curing would cause the polymer chains to tighten, resulting in smaller free volume or an increase in the membrane selectivity. Therefore, a lower flux was delivered by the membrane. The optimal curing temperature was established then to be 50 °C.

**Figure 7:**
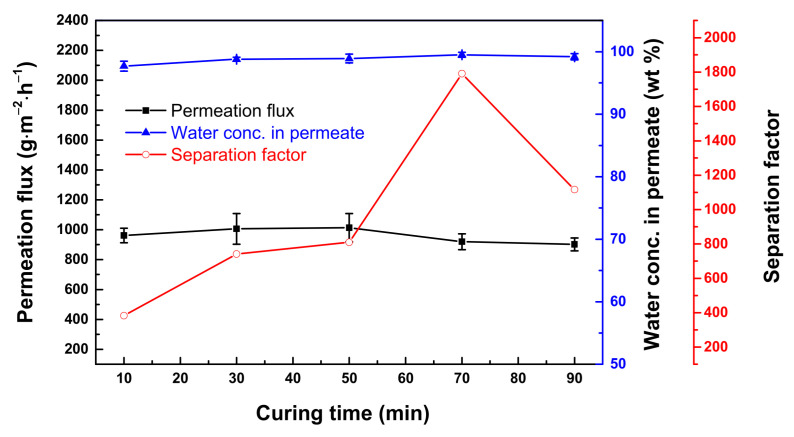
Effect of curing time on membrane performance. Feed = 90 wt.% aqueous THF solution; feed temperature = 25 °C. Interfacial polymerization conditions: 10 wt.% ethanol; membrane curing temperature = 50 °C.

Curing time also affects membrane performance [42]. Figure 7 plots the membrane performance as a function of curing time of 10 to 90 min. The optimal curing time was 30 min, wherein the permeation flux was 1006 ± 103 g·m^−2^·h^−1,^ and the water concentration in the permeate was 98.8 ± 0.3 wt.% (α_A/B_ = 741). At a time longer than 30 min, changes in the permeate concentration were no longer significant. This means that 30 min was enough for the densification of the thin-film polyurea layer. In this context, changes in the surface morphology (Figure S3) were not noticeable anymore.

## 4. Conclusions

A TFC polyurea membrane was fabricated through the interfacial polymerization reaction between PEI and XDI on an mPAN support for the dehydration of an aqueous THF solution. Alcohols that differed in the number of carbon were added as cosolvents to the aqueous solution containing PEI amine monomer. Adding an alcohol whose function was to swell the mPAN substrate favorably altered the membrane properties; in turn, the goal of achieving improved efficiency of pervaporation separation was realized. Based on the pervaporation experimental results, ethanol as a cosolvent was established to be the best swelling agent. The best separation efficiency provided a permeation flux of 1006 ± 103 g·m^−2^·h^−1^ and a permeate water concentration of 98.8 ± 0.3 wt.%.

## Figures and Tables

**Table 1:** Hydrophilicity of membranes.

Membrane	Water Contact Angle (^o^)
TFC	63.55 ± 2.07
TFC_Methanol_	61.40 ± 3.87
TFC_Ethanol_	63.96 ± 5.18
TFC_Isopropanol_	66.31 ± 2.57
TFC_tert-Butanol_	67.40 ± 2.86

## Data Availability

The data presented in this study are available on request from the corresponding author.

## Supplementary Materials

The following are available online at https://www.mdpi.com/article/10.3390/polym13081179/s1. Figure S1. Cross-sectional FESEM images: (a) mPAN; (b) TFC; (c) TFC_Methanol_; (d) TFC_Ethanol_; (e) TFC_Isopropanol_; and (f) TFC_tert-Butanol_, Figure S2. Surface FESEM images at different curing temperatures: (a) 30 °C; (b) 50 °C; (c) 70 °C; (d) 90 °C; and (e) 110 °C, Figure S3. Surface FESEM images at different periods of curing time: (a) 10 min; (b) 30 min; (c) 50 min; (d) 70 min; and (e) 90 min.
