# Supplementary material for: Cosolvent-Driven Interfacial Polymerization for Superior Separation Performance of Polyurea-Based Pervaporation Membrane
Source: Polymers (Basel). 2021 Apr 7;13(8):1179. doi: 10.3390/polym13081179 (PMC8067614; doi:10.3390/polym13081179)
Supplement: Supplementary file 1 [file polymers-13-01179-s001.pdf]

Cosolvent-Driven Interfacial Polymerization for Superior Separation Performance of Polyurea-based Pervaporation Membrane

Manuel Reyes De Guzman [1], Micah Belle Marie Yap Ang [2], Shu-Hsien Huang [3,2,*], Fang-Chi Hu [3], Yu-Hsuan Chiao [4], Hui-An Tsai [2] and Kueir-Rarn Lee [2,5]

[1] Material Corrosion and Protection Key Laboratory of Sichuan Province, School of Materials Science and Engineering, Sichuan University of Science and Engineering, Zigong 643000, China; manuelrdg@yahoo.com
[2] R&D Center for Membrane Technology and Department of Chemical Engineering, Chung Yuan Christian University, Taoyuan 32023, Taiwan; mbmyang@gmail.com (M.B.M.Y.A.); huian@cycu.edu.tw (H.-A.T.); krlee@cycu.edu.tw (K.-R.L.)
[3] Department of Chemical and Materials Engineering, National Ilan University, Yilan 26047, Taiwan; fangci@hotmail.com
[4] Department of Chemical Engineering, University of Arkansas, Fayetteville, AR 72701, USA; ychiao@uark.edu
[5] Research Center for Circular Economy, Chung Yuan Christian University, Taoyuan 32023, Taiwan
* Correspondence: huangsh@niu.edu.tw

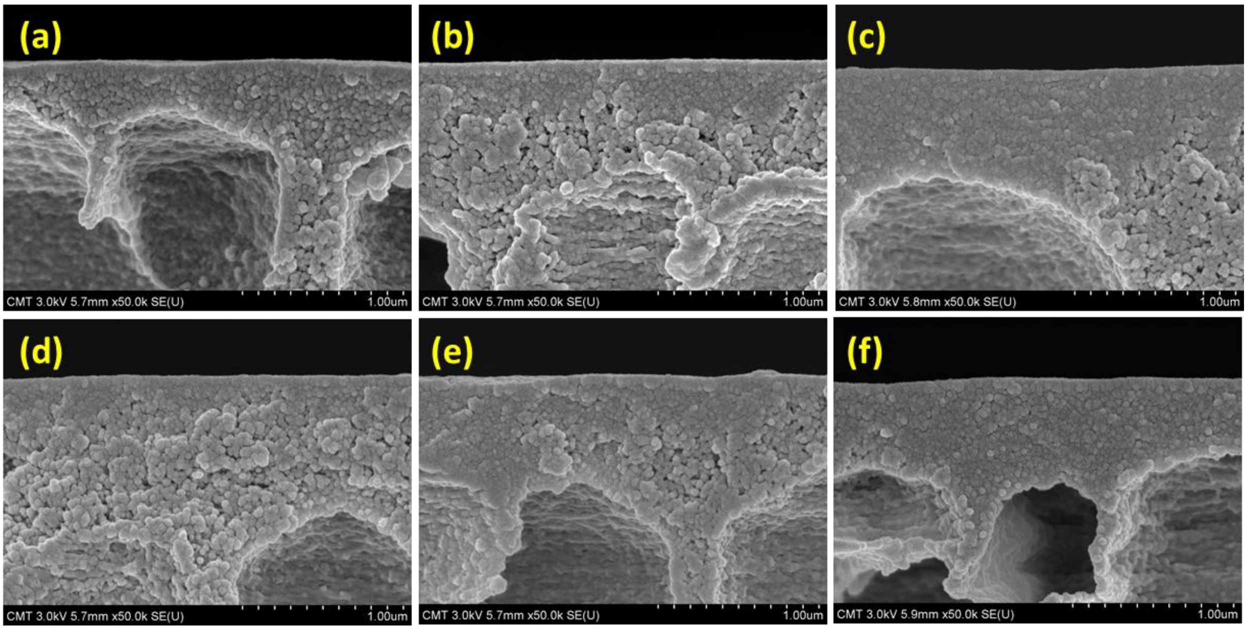

Figure S1. Cross-sectional FESEM images: (**a**) mPAN; (**b**) TFC; (**c**) $TFC_{Methanol}$; (**d**) $TFC_{Ethanol}$; (**e**) $TFC_{Isopropanol}$; (**f**) $TFC_{tert-Butanol}$.

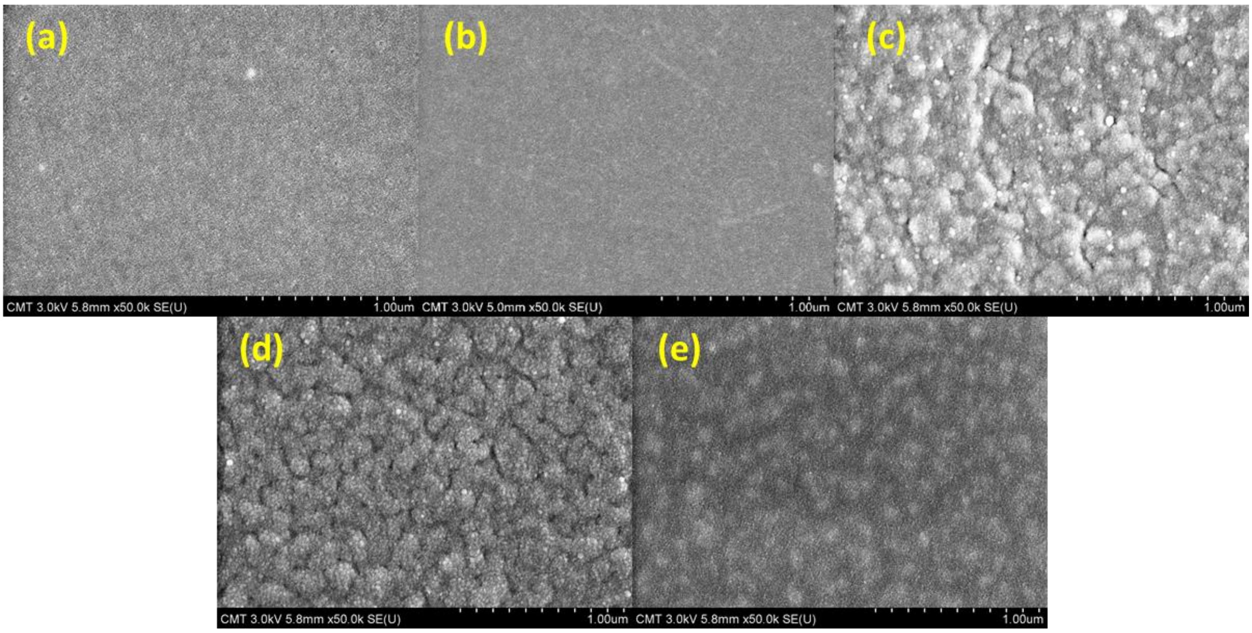

Figure S2. Surface FESEM images at different curing temperatures: (**a**) 30 °C; (**b**) 50 °C; (**c**) 70 °C; (**d**) 90 °C; (**e**) 110 °C.

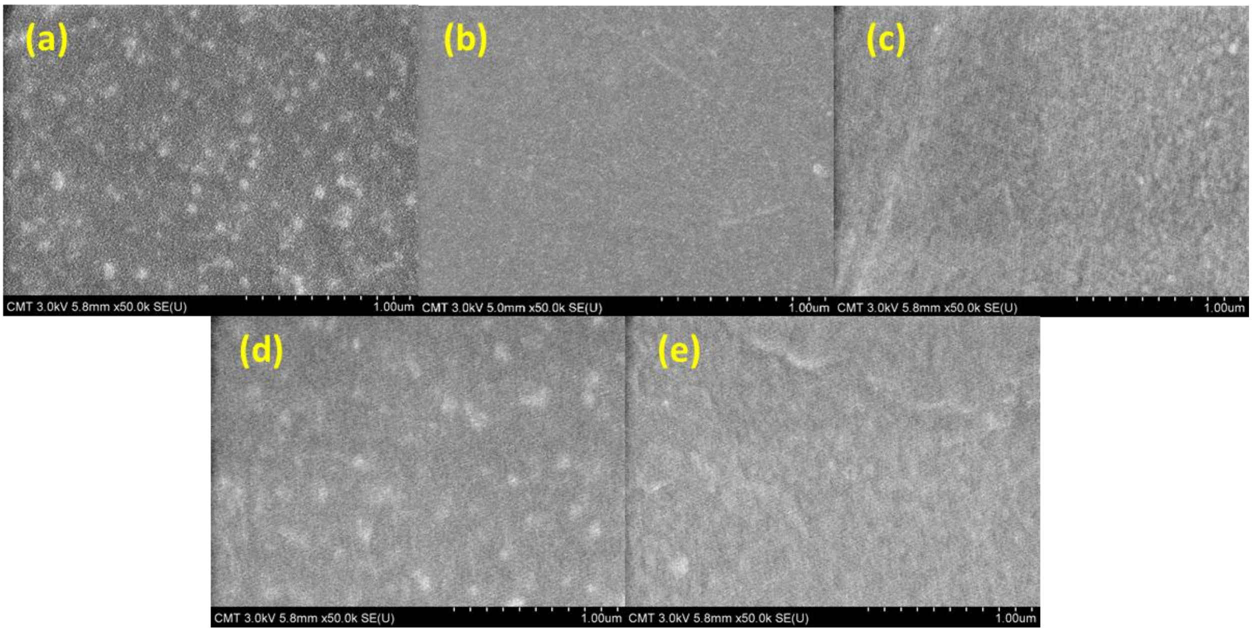

Figure S3. Surface FESEM images at different periods of curing time: (**a**) 10 min; (**b**) 30 min; (**c**) 50 min; (**d**) 70 min; (**e**) 90 min.
